# *Ehrlichia* species in pond-farmed leeches (*Hirudinaria* sp.) in Hubei Province, China

**DOI:** 10.1371/journal.pone.0215082

**Published:** 2019-04-08

**Authors:** Shu-Han Zhou, Xiao Xiao, Yi-Na Sun, Xiao-Hui Xu, Xin Ding, Si-Yi Zhang, Min Zhang, Wen-Liang Lv, Qing-Hua Gao

**Affiliations:** 1 Clinical College, Hubei University of Chinese Medicine, Wuhan, Hubei, China; 2 Lab Animal Research Center, Hubei University of Chinese Medicine, Wuhan, Hubei, China; 3 College of Basic Medicine, Hubei University of Chinese Medicine, Wuhan, Hubei, China; Johns Hopkins University, UNITED STATES

## Abstract

Leeches are frequently used in traditional Chinese medicine. However, they are potentially hazardous to human and animal health by transmitting several pathogens. Studies of diseases transmitted by leeches are scarce. The purpose of this study was to analyze the pathogens carried in pond-farmed medicinal leech in China. Leeches were collected from 6 farms in Hubei Province in central China. DNA was extracted from the internal organ of leeches to analyze the origin of blood meal. Leech genera were confirmed through amplification of 18S rRNA and mitochondrial gene cytochrome oxidase I (*COI*) gene by PCR and host animal species were identified through amplification of mitochondrial cytochrome *b* gene. Species of *Ehrlichia* in the leech specimens were screened with PCR using specific primers. PCR amplification and DNA sequencing showed that 620 leeches were *Hirudinaria* sp. *Ehrlichia* DNA was detected in 39 specimens from 2 farms. We obtained a total of 65 sequences of the *cytB* gene from 620 leech internal organ samples including sequences of human (n = 5), rat (n = 1), domestic pig (n = 10), duck (n = 23), goose (n = 12) and buffalo (n = 14). Phylogenetic analysis of the *rrs* and *groEL* gene sequences showed that *Ehrlichia* detected in the study were closely related to *Ehrlichia* sp. in ticks from Korea and Japan. To the best of our knowledge, this is the first report on *Ehrlichia* DNA being detected from leeches. Our findings provided new data on *Ehrlichia* spp. and farmed leech species in China.

## Background

Various leech species have been used worldwide in complementary medicine for centuries. Leeches secrete a complex mixture of different pharmacologically and biologically active substances into the wound while feeding[1]. Previous studies indicated that mammalian viruses, bacteria and bacteriophages persisted in the gut of leeches in large numbers for 23 weeks to more than 6 months [2, 3]. Leeches fed on the blood of different wild animals might be expected to bring about severe diseases by transmitting infectious agents that cause erysipelas (*Streptococcus* sp.), syphilis (*Treponema pallidum*), tetanus (*Clostridium tetani*), hog cholera (hog-cholera virus), and hospital wound infection (*Aeromonas hydrophila*) [2, 4–6]. Both *Bartonella* sp. and *B*. *grahamii* were detected from DNA extracted from terrestrial leeches (*Haemadipsa rjukjuana*)[7]. Leeches (*Haemadipsida* spp.) in Laos were reported as further potential vectors for *Rickettsia* infections[8]. *Rickettsia* DNA was also detected in field-collected specimens of *Torix tukubana*, *Torix tagoi* and *Hemiclepsis marginata*. Eggs produced by infected females of *T*. *tagoi* and *H*. *Marginata* were all tested *Rickettsia*-positive[9]. *Ozobranchus* (turtle leech) were also reported as a potential mechanical vector for the fibropapilloma-associated turtle herpesvirus[10]. Reports have also been published on experimental infection of leeches by classical swine fever virus, bovine parvovirus, feline calicivirus, equine arteritis virus, equine herpesvirus type 1, and infectious viruses were successfully reisolated from the leeches’ abdominal cavity blood at 23–29 weeks after inoculation[2]. Previous surveys over infectious agents transmitted by leeches in China were very limited. Therefore, the aim of this study was to investigate the prevalence of *Ehrlichia* in the pond-farmed leeches in the region of Hubei Province, central China.

## Methods

### Leech samples

**Fig 1:**
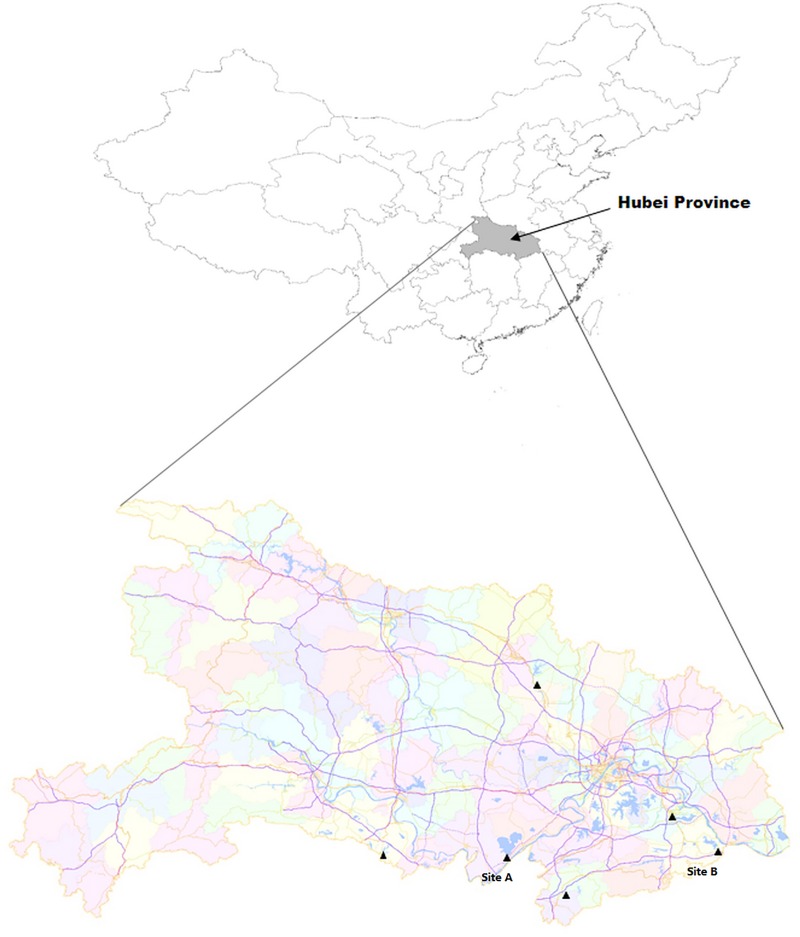
Geographic location of Hubei Province, China. Collection sites in this study are marked as triangles.

A total of 620 leeches were collected from outdoor leech farms in 6 different sites in Hubei Province (Fig 1) from June to September in 2018. Leeches were collected by net trapping with porcine blood clot as bait. The leeches were preserved in 70% ethanol and DNA extraction was performed with the QIAamp DNA Mini Kit (Qiagen, Hilden, Germany), DNA concentration and purity were measured with an absorbance ratio of 260 to 280 nm by using DeNovix DS-11 spectrophotometer (DeNovix, Wilmington, DE, USA) and were stored at -20°C until used. Mean quantity of DNA obtained in the internal organ of leeches was 43.6 ± 7.9 ng/μL and the 260/280nm ratio of all samples were 1.71 ± 0.16. Leech species was identified through the amplification of mitochondrial *COI* and 18S rRNA genes by PCR[11].

### PCR amplification of *Ehrlichia* and analysis of the host animals

For *Ehrlichia*, nested PCR amplifications of *rrs*, heat shock protein gene (*groEL*) and citrate synthase gene (*gltA*) were performed [12–14]. For analysis of the host animals which were fed by the leeches, the mitochondrial DNA cytochrome *b* gene was amplified by conventional PCR [15, 16]. The primers used for PCR amplification are listed in Table 1. All steps were performed in separate rooms to avoid contamination and distilled water was used as negative control.

**Table 1:** Nucleotide sequences of primers and conditions used for PCR amplification.

Organisms	PCR method	Primer	Primer sequences	Target gene	Annealing temp (°C)	Amplicon size (bp)	References
Leech	PCR	LCO 1490	GGTCAACAAATCATAAAGATATTGG	Mitochondrial *COI*	44	710	[17]
		HCO 2198	TAAACTTCAGGGTGACCAAAAAATCA				
Leech	Nested PCR	A	AACCTGGTTGATCCTGCCAGT	18S rRNA	47	1200	[18]
		B	TGATCCTTCCGCAGGTTCACCT				
		Y	CAGACAAATCGCTCC		47	926	[18]
		C	CGGTAATTCCAGCTC				
*Ehrlichia*	Nested PCR	EC9	TACCTTGTTACGACTT	*rrs*	52	1462	[13]
		EC12A	TGATCCTGGCTCAGAACGAACG				
		HF51f	AAGTCGAACGGACAATTACC		55	923	[14]
		HF954r	GTTAGGGGGATACGACCTTC				
		5gltA-out	GGCATTTTTCCTGATGTGCATGAT	*gltA*	60	897	[12]
		3gltA-out	ATACCATTGAGCCGACCAGCC				
		5gltA-in	AGCAGTGTCTCAAATTGCAGG		56	426	[12]
		3gltA-in	ATCCTATGGCCAAAACCCATTA				
		5GroELout	GTACGGCTGGACCTAAAGGA	*groEL*	60	701	[12]
		3GroELout	AGTGCTGAGAGCTTCACCTTC				
		5GroELin	ATGGGGCACCAGAAGTTACA		56	422	[12]
		3GroELin	CCACGATCAAATTGCATACCATCA				
Host animals	PCR	L14816	CCATCCAACATCTCAGCATGATGAAA	Mitochondrial *CytB*	50	358	[15]
		H15173	CCCCTCAGAATGATATTTGTCCTC				
		L14841	AAAAAGCTTCCATCCAACATCTCAGCATGATGAAA		50	450	[16]
		H15149	AAACTGCAGCCCCTCAGAATGATATTTGTCCTCA				

PCR products were separated with 1.2% agarose gel electrophoresis and visualized by ethidium bromide staining under UV. PCR products with expected sizes were excised from gels and extracted using a Gel Extraction Kit (Promega, Madison, WI), which were then cloned into the pMD19-T vector (TaKaRa, Shiga, Japan). Recombinant clones were selected using blue-white screening method. Three recombined plasmids for each PCR product were selected and cultured at 37°C for 10–15 hrs in shaking incubators. Tsingke plasmid DNA prep kit PM0201-200 (Tsingke, Wuhan, HB) was used in the isolation of plasmid DNA, all according to the manufacturer’s instructions.M13F-47, M13R-48 Universal Primers were used for Sanger dideoxy sequencing in TingKe Biotech Company (Wuhan, China) on both strands.

### Phylogenetic analysis

All sequences were searched using BLAST in the GenBank database (http://blast.ncbi.nlm.nih.gov/Blast.cgi). After alignment by ClustalW with MEGA 7.0, the evolutionary models for our datasets were estimated using jModeltest2 [19, 20]. Phylogenetic trees were constructed using the Maximum Likelihood method in MEGA7.0, and the robustness of the trees was tested with 1,000 bootstrap replications.

### Statistical analysis

The infection rates of pathogens in different months were calculated and 95% confidence intervals were estimated by *U*-test. Statistical analysis was performed using R version 3.4.2 (https://www.r-project.org/).

## Results

### Identification of leech species

**Fig 2:**
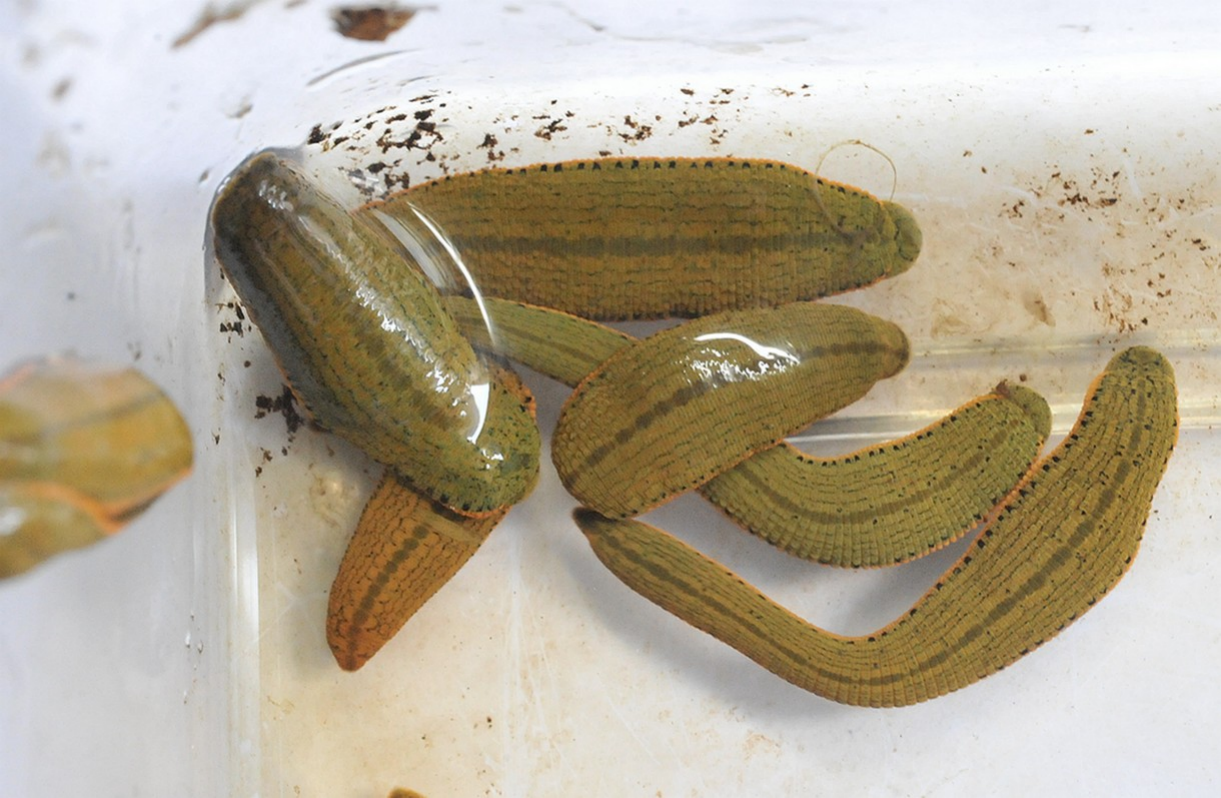
Medicinal leeches (*Hirudinaria* sp.) collected in this study.

**Fig 3:**
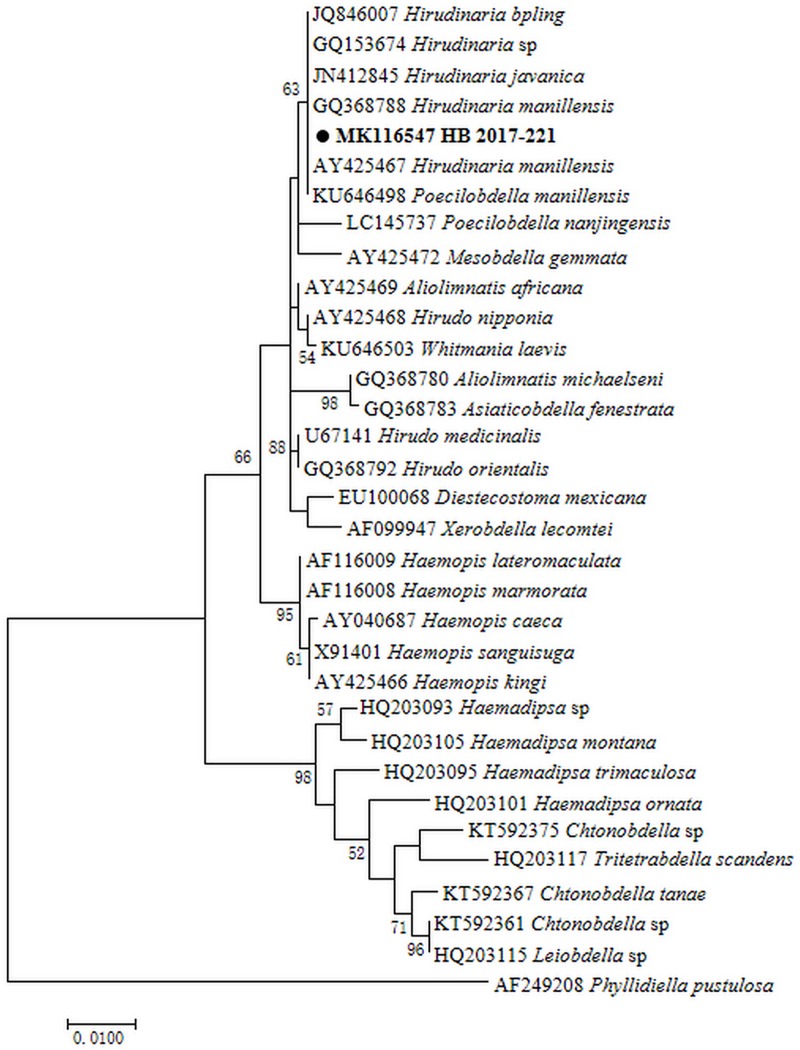
Maximum likelihood phylogenetic tree of leech species based on the 18S rRNA gene. The tree was constructed with the 18S rRNA sequences (926bp) by using the Kimura 2-parameter model with MEGA 7.0 (http://www.megasoftware.net); we rooted using *Phyllidiella pustulosa* sequence from Genbank and calculated bootstrap values with 1,000 replicates. The representative sequence of leeches in this study are in bold print and marked by circle. Scale bar indicates nucleotide substitutions per site.

**Fig 4:**
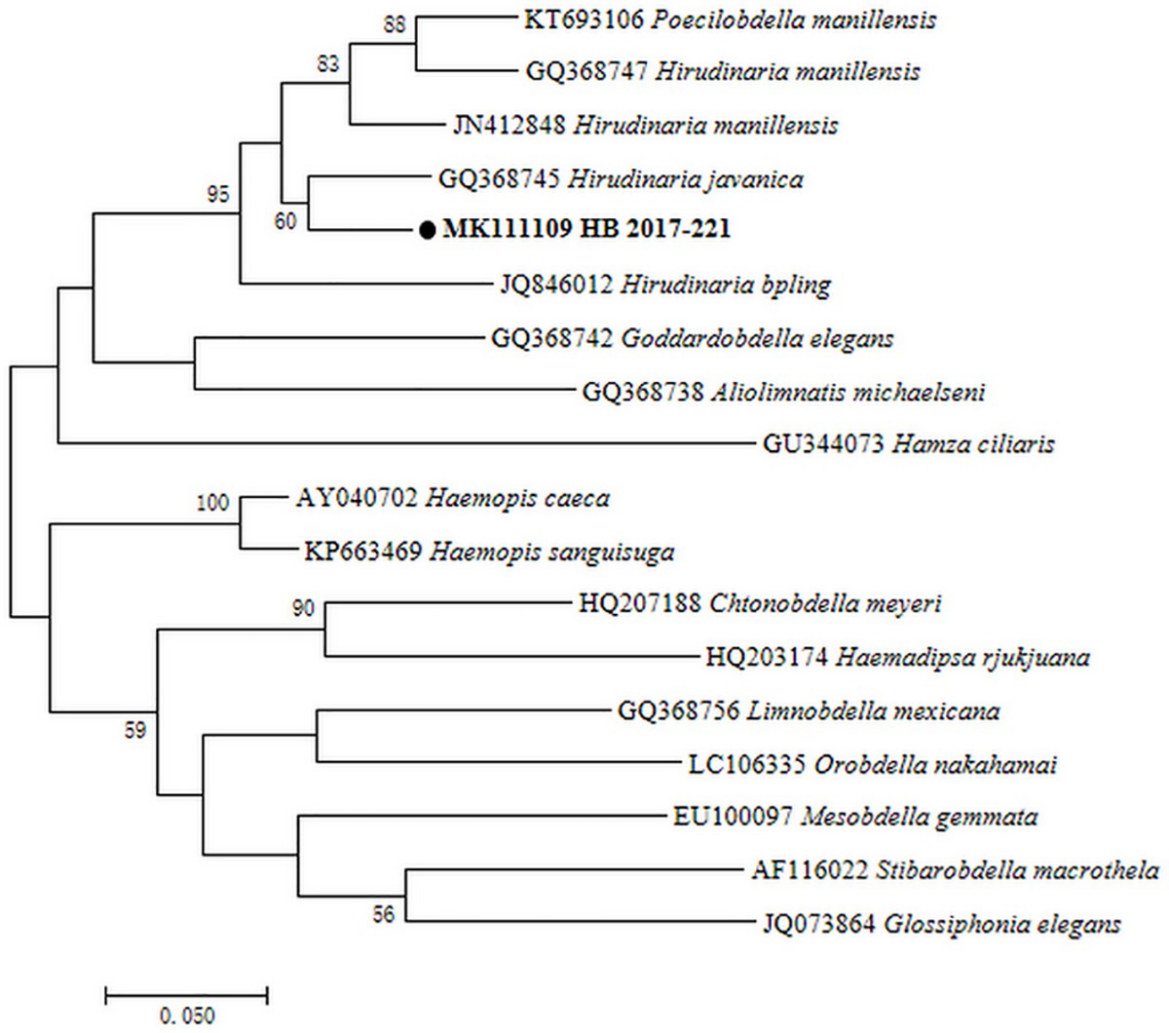
Maximum likelihood phylogenetic tree of leech species based on the *COI* gene. The tree was constructed with the *COI* sequences (682bp) by using the Kimura 2-parameter model with MEGA 7.0 (http://www.megasoftware.net); we calculated bootstrap values with 1,000 replicates. The representative sequence of leeches in this study are in bold print and marked by circle. Scale bar indicates nucleotide substitutions per site.

Leech specimen from all 6 farms showed high similarity morphologically (Fig 2). Thirty 18S rRNA sequences obtained from 30 randomly selected representative specimens were 99.6–100% identical to each other. A total of 22 *COI* sequences were amplified from these specimens and showed 99.2–100% similarity to each other. BLAST analysis showed leech genus in this study were closely related to *Hirudinaria* sp. from Malaysia and Vietnam with 98.6–99% sequence similarity in 18S rRNA (GenBank: GQ153674) and 93.4–93.6% in *COI* (GenBank: GQ368747). Phylogenetic analysis indicated the representative specimens clustered with *Hirudinaria* sp. from Southeast Asia both in 18S rRNA and *COI* (Figs 3 and 4).

### *Ehrlichia* in leeches

The PCR amplification of the 16s rRNA (*rrs*) sequences resulted in detection of *Ehrlichia* from 39 of the 620 leech specimens. All 39 sequences were highly identical (99.6–100%) to each other. Three representative sequences exhibited a 99–100% similarity with *Ehrlichia* sp. (GenBank: GU075697) in *Haemaphysalis longicornis* from Jeju Island, South Korea. All *rrs* positive samples were further confirmed through amplification of *gltA* and *groEL*. The *gltA* gene amplifications were unsuccessful, and we obtained 22 sequences of *groEL* eventually. All 22 *groEL* sequences were 99.8–100% identical to each other. BLAST analysis of the representative *groEL* sequence indicated it was 99% homologous to *Ehrlichia* sp. (GenBank: HQ697590) detected in ticks from Yonaguni Island, Japan.

**Fig 5:**
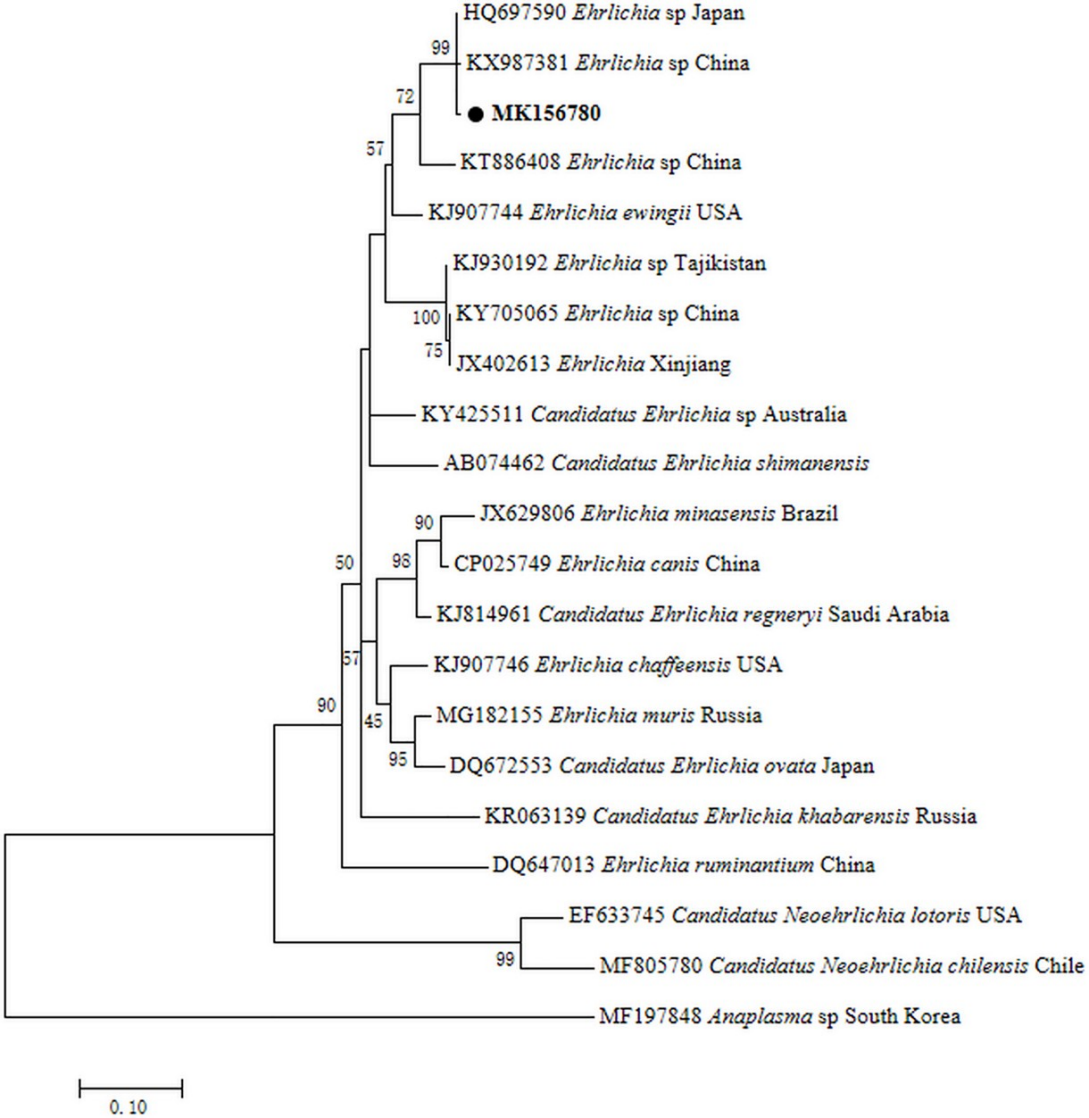
Maximum likelihood phylogenetic tree based on the *rrs* gene of *Ehrlichia*. The tree was constructed with the *rrs* sequences (923bp) by using the general time-reversible model with MEGA 7.0 (http://www.megasoftware.net); we rooted using *Anaplasma phagocytophilum* sequence from Genbank and calculated bootstrap values with 1,000 replicates. The representative sequences obtained in this study are in bold print and marked by circles. Scale bar indicates nucleotide substitutions per site.

**Fig 6:**
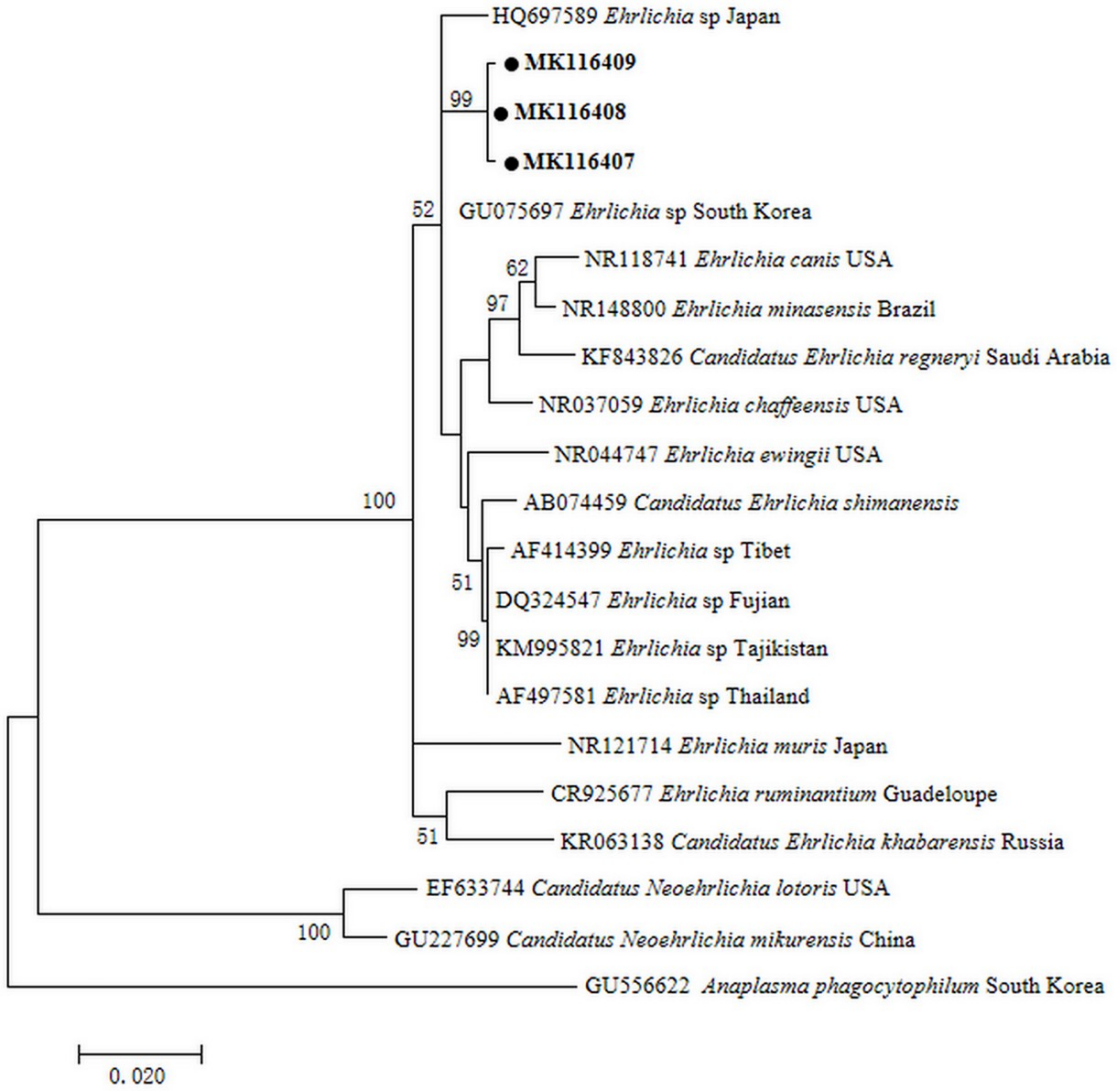
Maximum likelihood phylogenetic tree based on the *groEL* gene of *Ehrlichia*. The tree was constructed with the *groEL* sequences (422bp) by using the Tamura 3-parameter model with MEGA 7.0 (http://www.megasoftware.net); we rooted using *Anaplasma* sp. sequence from Genbank and calculated bootstrap values with 1,000 replicates. The representative sequence obtained in this study are in bold print and marked by circle. Scale bar indicates nucleotide substitutions per site.

Phylogenetic analysis based on the *rrs* showed that *Ehrlichia* sequences detected from leeches formed a clade together with uncultured *Ehrlichia* species from ticks in Jeju Island and Yonaguni Island (Fig 5). Phylogenetic analysis of *groEL* gene also indicated that *Ehrlichia* detected in leeches clustered together with uncultured *Ehrlichia* sp. (Fig 6) that was previously documented in ticks from Jeju Island and Yonaguni Island. The results indicated that the *Ehrlichia* from leeches appeared to be the same strain of *Ehrlichia* existing in ticks from South Korea and Japan. The infection rates of *Ehrlichia* in leeches from two farms (Site A and B) in different months were presented in Table 2.

**Table 2:** Infection rate of *Ehrlichia* in leeches in different months from two farms.

	Pa(n)	Pb(n)	Total	Prevalence (95%CI) (%)
June	2(12)	4(31)	6(43)	14(4–24)
July	9(36)	5(52)	14(88)	16(8–24)
Aug	2(33)	11(49)	13(82)	16(8–24)
Sep	3(65)	3(53)	6(118)	5(1–9)

P_a_: Number of positve samples in site A; P_b_: Number of positve samples in site B

95%CI: 95% confidence interval of prevalence of *Ehrlichia* in leeches estimated by *U*-test.

### Identification of leech host animals

The host animals of leech were identified by amplification of the mitochondrial DNA cytochrome *b* gene. A total of 65 sequences were successfully obtained from 620 leeches. The 65 sequences were identified as human (n = 5), rat (n = 1), domestic pig (n = 10), Chinese muscovy duck (n = 23), Chinese goose (n = 12), water buffalo (n = 14) (Table 3). About 46.2% of the sequences were mammalians and 53.8% were domestic waterfowls. *Ehrlichia* species were detected from 3 leeches with blood meals of Asian water buffalos and 2 leeches with blood meals of domesticated pigs.

**Table 3:** Animal hosts of leeches determined by PCR using mtDNA cytochrome *b* gene from leeches.

Detected hosts	Scientific names	Category	No. of positive samples
Human	*Homo sapiens*	Mammal	5
Rat	*Rattus norvegicus*	Mammal	1
Domestic pig	*Sus scrofa*	Mammal	10
Chinese muscovy duck	*Cairina moschata*	Bird	23
Chinese goose	*Anser cygnoides*	Bird	12
Water buffalo	*Bubalus bubalis*	Mammal	14
**Total**			**65**

## Discussion

Medicinal leech therapy has a long history in complementary medicine. However, studies recently suggested that leeches might be promising candidates as vectors of pathogens. To the best of our knowledge, this is the first report of *Ehrlichia* DNA being detected from leeches. Previous studies demonstrated that terrestrial leeches feeding on humans and animals contained *Bartonella* spp. in South Korea, and a woman in Laos was confirmed *Rickettsia felis* infection after being bitten by a terrestrial leech *(Haemadipsida* spp.) [7, 8]. These results suggest that leeches may harbor zoonotic bacteria and leech therapy pose potential risks of patients becoming infected with zoonotic agents. The genus *Ehrlichia* consists of several species of obligate Gram-negative intracellular bacteria that is transmitted to vertebrates by tick bites [21]. *E*. *chaffeensis* is the major etiologic agent of human monocytotropic ehrlichiosis (HME), and canine monocytic ehrlichiosis (CME) is a serious and sometimes fatal tick-borne disease in dogs caused by *E*. *canis*[22, 23]. *E*.*ewingii*, a veterinary pathogen associated with granulocytic ehrlichiosis in dogs was also identified in four patients from Missouri in 1999 and thus became the least known agent of the human ehrlichioses[24]. The spread and maintenance of *Ehrlichia* involve complex zoonotic systems including vectors and persistently infected vertebrate reservoirs. Severe life-threatening illnesses, such as HME and heartwater, occur mostly in incidental hosts while infections appear to be subclinical in natural hosts. Isolation of *E*.*chaffeensis* from wild white-tailed deer (*Odocoileus virginianus*) confirmed their role as natural reservoir hosts[25]. Previous studies also indicated African ruminants including black wildebeest, African buffalo and eland as proposed reservoirs of *E*.*ruminantium*[26]. Rodents are the natural reservoir hosts of *E*.*muris* and several carnivores including red fox, may play a role in the epidemiology of canine ehrlichiosis [27–30]. Recently, histopathology and PCR analysis confirmed *Ehrlichia* infection in goats from Wuhan, Hubei Province, China[31]. Extensive diversity of Rickettsiales bacteria in multiple ticks and mosquito species was also recorded in the same region. *Ehrlichia* DNA was mainly found in *Rhipicephalus microplus* and *Haemaphysalis longicornis* ticks in Hubei Province, and *Ehrlichia* bacteria has been detected in each life stage of mosquitoes, suggesting that *Ehrlichia* may be maintained in mosquitoes through both transstadial and transovarial transmission[32, 33]. However, few studies have been conducted to evaluate the infection of Ehrlichiosis in human and wild animals in this region. The number of outdoor leech farms has been increasing in the past few years. However, leech farming in China is unregulated, outdoor farming facilities and environments vary greatly, from natural ponds, cement pool, net cage to net surrounded earthen pond. Live leeches have been freely sold online and shipped alive nationwide. All leeches obtained in this study were identified to be *Hirudinaria* species, which is a non-native, but commonly farmed genus in central China[34]. Zoonotic bacteria and viruses acquired from previous host via sucking can remain infectious in the leech gut for months [2]. While nested PCR and sequencing of amplified DNA fragments confirmed the presence of *Ehrlichia* in leeches, detection of *Ehrlichia* DNA in the leeches may represent residual *Ehrlichia* DNA from hosts’ blood. Since pond-farmed leeches occasionally feed on the blood of animals that likely carry various pathogens, it is necessary to isolate and characterize *Ehrlichia* from leeches with macrophage-derived cells to further understand the role of leeches in *Ehrlichia* ecology. Infection rate of *Ehrlichia* in leeches in different seasons might be biased due to the limited sample size. Whether leeches obtained *Ehrlichia* from the feeding animal hosts or they persistently carried *Ehrlichia*, and the possibilities of vector-host transmission, vertical or horizontal transmission of *Ehrlichia* in leeches also need to be further confirmed. However, to our knowledge, this is the first report of *Ehrlichia* DNA being detected from leeches. Our study provided new evidence on the potential role of leeches in the transmission of *Ehrlichia*. To further assess the prevalence and possible transmission of *Ehrlichia* by leeches, more extensive research is needed.

## Conclusion

We first time detected *Ehrlichia* DNA in pond-farmed leeches (*Hirudinaria* sp.) in Hubei Province, China. Phylogenetic analysis based on the *rrs* and *groEL* genes showed that *Ehrlichia* sequences detected from leeches formed a clade together with uncultured *Ehrlichia* species from ticks in Jeju Island, ROK and Yonaguni Island, Japan. Our findings provided new data on *Ehrlichia* spp. and farmed leech species in China.

## Supporting information

S1 Table Geographic coordinates of 6 collection sites in this study. (DOCX)
