# Supplementary material for: Ehrlichia species in pond-farmed leeches (Hirudinaria sp.) in Hubei Province, China
Source: PLoS One. 2019 Apr 8;14(4):e0215082. doi: 10.1371/journal.pone.0215082 (PMC6453479; doi:10.1371/journal.pone.0215082)
Supplement: S1 Table — (DOCX) [file pone.0215082.s001.docx]

| **Collection Sites** | **Longitude** | **latitude** |
| --- | --- | --- |
| 1 | 114°56'E | 30°05'N |
| 2 | 112°05'E | 29°45'N |
| 3 | 114°01'E | 29°31'N |
| 4 | 113°35'E | 31°26'N |
| 5 (Site A) | 113°16'E | 29°44'N |
| 6 (Site B) | 115°18'E | 29°50'N |

**S1 Table. Geographic coordinates of 6 collection sites in this study**
